# Association of self-reported snoring with decreased retinal thickness and vessel density

**DOI:** 10.3389/fphys.2022.917808

**Published:** 2022-08-05

**Authors:** Yunfan Xiao, Keai Shi, Chunmei Li, Kai Yang, Xiaoxuan Zhu, Binbin Su, Ying Ju, Fan Lu, Jia Qu, Ming Li, Lele Cui

**Affiliations:** Eye Hospital and School of Ophthalmology and Optometry, National Clinical Research Center for Ocular Diseases, Wenzhou Medical University, Wenzhou, Zhejiang, China

**Keywords:** optical coherence tomography, optical coherence tomography angiography, self-reported snoring, macular microcirculation, macular structure

## Abstract

**Purpose:** Self-reported snoring has been reported to influence nerves and vessels. However, there are few direct evidences of snoring related to nerves and microvessels defects. Therefore, we evaluated the association of self-reported snoring with retinal structure and microcirculation.

**Methods:** A total of 2,622 participants were recruited from the Jidong eye cohort study (JECS). Physical examinations, laboratory tests, and questionnaires were recorded. We also used optical coherence tomography (OCT) and optical coherence tomography angiography (OCTA) to assess the retinal structure and microvascular network. Snoring was defined as “never,” “occasionally,” and “frequently or more severe” according to self-reported frequency.

**Results:** The prevalence of snoring were 84.6% (*n* = 983) and 45.0% (*n* = 657) in males and females, respectively. Compared with never snoring group, the retinal thickness increased in “occasionally” (*p* < 0.001) and “frequently or more severe” groups (*p* = 0.001), while no difference was found between snoring groups (*p* = 0.14). Superficial retinal capillary plexus (RCP) vessel density was lower in “frequently or more severe” group than in “never” (*p* < 0.001) and “occasionally” snoring groups (*p* < 0.001). After adjusting for confounders, “frequently or more severe” snoring was significantly associated with thinner total retinal thickness [*β* = −2.79 (95% CI: −5.27, −0.30)] and lower superficial RCP vessel density [*β* = −0.71 (95% CI: −1.19, −0.23)].

**Conclusion:** Our research showed self-reported snoring was associated with thinner retinal thickness and lower superficial RCP vessel density. The findings of our study emphasize the need for self-reported snoring assessments in determining retinal structure and microcirculation impairment.

## Introduction

Snoring is a common harmful sleep habit that is characterized as breathing sounds produced by the vibration of the pharyngeal wall and its posterior associated structures (de Felício et al., 2018). Several studies reported the prevalence of snoring ranged from 50 to 94% in adults (Xiong et al., 2016; Zhang et al., 2017; De Meyer et al., 2019; Rosen et al., 2019; Jiang et al., 2021). Snoring is also a common early symptom of obstructive sleep apnea (OSA) (Young et al., 2002), and OSA has been confirmed to be associated with diabetes (Reutrakul and Mokhlesi, 2017), metabolic disorders (Gaines et al., 2018), cardiovascular events (Bradley and Floras, 2009) and all-cause mortality (Lechat et al., 2021). Many snorers are not diagnosed with OSA, and snoring without an OSA diagnosis has long been considered a social nuisance and brought relevant burden (Deary et al., 2014; Brockmann et al., 2015). Recently, a growing number of studies have shown that snoring is also a risk factor for the aforementioned diseases and is independent of OSA (Lee et al., 2008; Zou et al., 2019; Taylor et al., 2021). The vessel vibration caused by snoring may induce arterial endothelial injury and promote the formation of atherosclerosis (Li et al., 2012; Behl et al., 2014). Snoring with airway stenosis and hypoxia may lead to nerve impairment (Whyte and Gibson, 2020). However, there was no direct evidence of peripheral nerve and microvascular changes in people who snore.

The retina reflects ocular health status, and previous studies have observed changes of the peripheral nervous system and vascular diseases in the retina (Wang et al., 2018; Wylęgała, 2018; Tang et al., 2020; István et al., 2021). In recent years, optical coherence tomography (OCT) technology has allowed a high-resolution and noninvasive visualization of the retinal structure. Optical coherence tomography angiography (OCTA) develops from OCT and focuses on vascular imaging, which can quantitatively analyze the retinal microcirculation network. These two technologies have been widely used and recognized in ophthalmology. A few studies have found ocular changes in patients with OSA using OCT and OCTA (Lee et al., 2019; Venkatesh et al., 2021). Nevertheless, to our knowledge, snoring related retinal changes have not yet been reported. Therefore, we aimed to investigate the association of self-reported snoring with retinal structure and microcirculation using OCT and OCTA in a large community-based population.

## Materials and methods

### Participants

The data in this study were derived from the Jidong eye cohort study (JECS), which was a community-based study that has been described in detail (Yang et al., 2020). In brief, the JECS was established in 2019 and aimed to assess ocular biological indicators and their association with cardiovascular and neurological diseases. A total of 3,377 participants with complete examination records were enrolled, leaving 2,622 for analysis after excluding individuals with ocular history or surgery (*n* = 19), cerebral/cardiovascular and neurological conditions (*n* = 102), missing questionnaires (*n* = 42), axial length ≥26 mm (*n* = 371), and substandard OCTA images (*n* = 221).

This study followed the tenets of the Declaration of Helsinki and received approval from the Ethics Committee of the Staff Hospital of the Jidong Oil-Field of Chinese National Petroleum. Informed consent was obtained from each participant.

### Baseline information

Baseline characteristics, including demographic data, clinical and laboratory examinations, and ocular biological parameters, were collected. The body mass index was calculated using weight (kg) divided by height squared (m^2^). Blood pressure was obtained with a digital automatic blood pressure monitor after adequate rest. Complete blood count, biochemistry and lipid profile were tested after at least 8 h of fasting. Participants were regarded as having hypertension, diabetes mellitus, and dyslipidemia if any of the following criterion was met: 1) self-reported history or 2) self-reported medication history or 3) clinical or laboratory examination (blood pressure ≥140/90 mmHg for hypertension, fasting glucose level ≥7.0 mmol/L for diabetes mellitus, serum triglyceride ≥1.76 mmol/L or low-density lipoprotein cholesterol ≥3.37 mmol/L or high-density lipoprotein cholesterol ≤1.04 mmol/L for dyslipidemia).

All participants had a complete record of ophthalmic examination. Best corrected visual acuity (BCVA) was assessed using a 5-meter-distance standard logarithmic visual acuity chart. Refractive error was represented with spherical equivalent (SE, sphere plus one-half cylinder), which was measured using an autorefractor (KR800; Topcon; Tokyo, Japan). Axial length was assessed by ocular biometry (Lenstar 900 Optical Biometer; Hagg-Streit, Koeniz, Switzerland). The ocular anterior segment was estimated with a slit-lamp biomicroscope, and the posterior segment was imaged with digital photography (CR2AF; Canon, Tokyo, Japan). All above-mentioned examinations were performed in 1 day.

### Questionnaire definition

Face-to-face interviews were conducted by trained research coordinators to collect questionnaire information. The frequency of snoring was assessed by the question “Do you snore when you sleep?” The individuals were grouped according to their responses (“never,” “occasionally,” “frequently or more severe”). Information on snoring was collected with the help of participant’s spouse or relatives. Nonsmokers and nondrinkers were identified by negative responses to the following questions: “Have you ever smoked” and “Have you ever drunk?”

### Optical coherence tomography and optical coherence tomography angiography assessment

**FIGURE 1:**
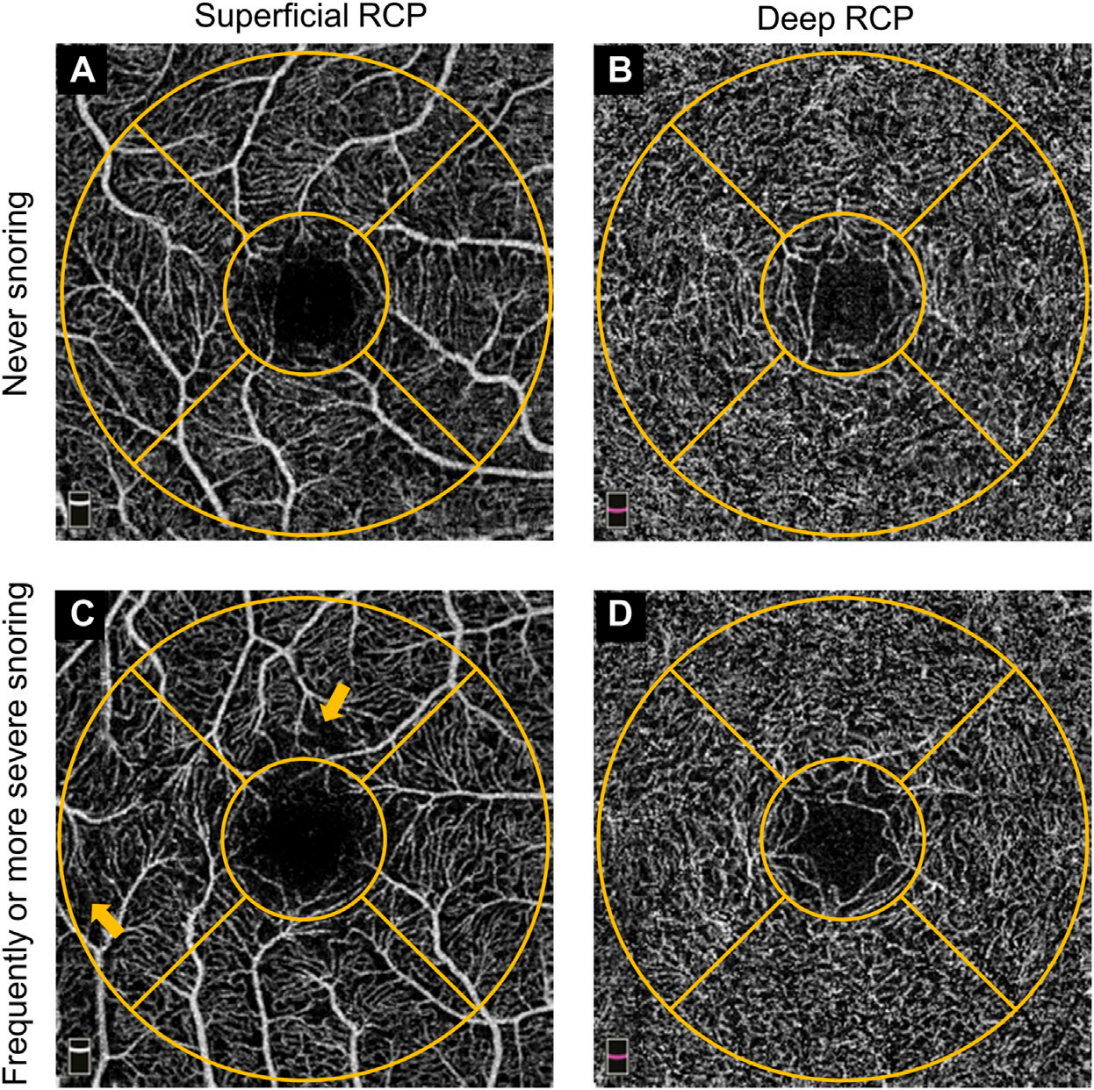
Representative OCTA images of self-reported never snoring and frequently or more severe snoring participants. **(A)**. Superficial RCP image from a never snoring participant. **(B)**. Deep RCP image from a never snoring participant. **(C)**. Superficial RCP image from a frequently or more severe snoring participant. **(D)**. Deep RCP image from a frequently or more severe snoring participant. The orange-colored concentric circle represented the ETDRS grid with 1 mm and 1–3 mm diameters, respectively. Origin arrows showed the superficial RCP vessel lost. OCTA, optical coherence tomography angiography; RCP, retinal capillary plexus; ETDRS, Early Treatment of Diabetic Retinopathy Study.

The evaluation process of the retinal structure and microcirculation was performed using the spectral-domain OCTA device (RTVue XR Avanti with AngioVue; Optovue, Inc., Fremont, CA, United States). Details of the procedures and technology has been previously provided (Laíns et al., 2021). In brief, this scanning and analysis system used low-coherence near-infrared light to image biological tissues and obtained retinal cross-sectional structural images and a list of *en face* vascular projections. The scan mode centered on the macula with 3 × 3 mm^2^ area and had 304 horizontal A-scans per vertical B-scan line. Completed images were further assessed by built-in software (Optovue, Inc., Fremont, CA, United States) to obtain correct retinal segmentation: the total retinal thickness was defined from the inner limiting membrane (ILM) to Bruch’s membrane (BM): retinal nerve fiber layer (RNFL), ganglion cell layer (GCL), inner plexiform layer (IPL), inner nuclear layer (INL), outer plexiform layer (OPL), external limiting membrane (ELM), photoreceptor layer (PR), retinal pigment epithelium (RPE), and BM. It was a complex tissue that contained retinal neuroepithelial layer, pigment epithelium, and the hyaline membrane. The superficial retinal capillary plexus (RCP) was from the ILM to the inner plexiform layer (IPL), and the deep RCP was from the IPL to the outer plexiform layer. The Early Treatment Diabetic Retinopathy Study (ETDRS) map was used to present the processed components, and the map contains a central circle and a concentric ring with diameters of 1 and 1-3 mm, respectively. The outer ring was continuously divided into four quadrants (temporal, superior, nasal and inferior). 3D-projection artifact removal algorithms (Optovue, Inc., Fremont, CA, United States) were used to remove superficial middle vessel signals from the deep layer. The accuracy of the measurement of retinal thickness and vessel density had been proved in previous studies. Figure 1 showed the representative OCTA images of the self-reported “never” snoring and “frequently or more severe” snoring.

Two trained ophthalmologists reviewed all OCT and OCTA images and excluded defective images if they met any of the following criteria: 1) incorrect segmentation; 2) obvious decentration misalignment; 3) serious motion artifacts; and 4) signal index <7. By standard, we included the right eye of each participant for analysis.

### Statistical analysis

Continuous variables were described as the mean [standard deviation (SD)]. Categorical variables were summarized using counts (percentage). One-way analysis of variance (ANOVA) and the χ2 test were applied to analyze the difference among self-reported snoring groups.

We employed a multivariable generalized linear model (GLM) to explore the association of snoring with retinal parameters, and confounders (age, sex, body mass index, education level, history of diabetes, hypertension, dyslipidemia, smoking and drinking, creatinine level, number of red blood cells, and hemoglobin level) were adjusted in this model, considering the statistical significance in baseline and clinical related or cared. Never snoring was set as a reference. Multicollinearity was checked using variance inflation factors. We also performed stratified analysis to determine the potential modification effects of hypertension. The association was presented as *β* coefficients with 95% confidence intervals (CI). A two-tailed P level <0.05 was defined as statistically significant. All analyses were performed using SPSS (version 26.0; IBM; NY, United States).

## Results

### Baseline characteristics

A total of 2,622 eligible participants were enrolled in this study, and the mean (SD) age was 44.0 (11.6) years. There were 1,162 men, and 983 (84.6%) of them reported different frequencies of snoring. There were also 657 (45.0%) females who snored. Compared with those in the “never” snoring group, participants with more frequent snoring were more likely to be smokers and drinkers. In addition, participants in the snore groups had a higher prevalence of hypertension, diabetes and dyslipidemia, and a higher BMI, serum creatinine level, red blood cell count and hemoglobin level. However, there was no significant difference in education among snore groups. Table 1 shows the baseline characteristics of the groups with different snoring frequencies.

**TABLE 1:** Baseline characteristics of eligible participants according to frequency of self-reported snoring.

Characteristic	overall (*n* = 2,622)	Self-reported snoring			*p* value	*P* value^a^	*P* value^b^	*P* value^c^
		never (*n* = 982)	occasionally (*n* = 1,470)	Frequently or more severe (*n* = 170)				
Age, mean (SD), years	44 (11.6)	42.3 (11.5)	44.8 (11.5)	47 (11.5)	<0.001	<0.001	<0.001	0.02
Gender					<0.001	<0.001	<0.001	<0.001
Female, No. (%)	1,460 (55.7%)	803 (81.8%)	644 (43.8%)	13 (7.6%)				
Male, No. (%)	1,162 (44.3%)	179 (18.2%)	826 (56.2%)	157 (92.4%)				
BMI, mean (SD), kg/m^2^	24.4 (3.5)	23.2 (3.0)	24.9 (3.5)	27 (3.6)	<0.001	<0.001	<0.001	<0.001
Hypertension, No. (%)	658 (25.1%)	157 (16.0%)	416 (28.3%)	85 (50.0%)	<0.001	<0.001	<0.001	<0.001
Diabetes, No. (%)	211 (8.0%)	41 (4.2%)	142 (9.7%)	28 (16.5%)	<0.001	<0.001	<0.001	0.006
Dyslipidemia, No. (%)	1,268 (48.4%)	375 (38.2%)	777 (52.9%)	116 (68.2%)	<0.001	<0.001	<0.001	<0.001
College or above, No. (%)	1798 (69.0%)	666 (68.3%)	1,022 (69.9%)	110 (65.1%)	0.38	0.42	0.41	0.20
Smoke, No. (%)	560 (21.4%)	81 (8.3%)	389 (26.6%)	90 (52.9%)	<0.001	<0.001	<0.001	<0.001
Drink, No. (%)	355 (13.6%)	65 (6.7%)	217 (14.9%)	73 (42.9%)	<0.001	<0.001	<0.001	<0.001
LDL, mean (SD), mmol/L	2.1 (0.8)	2.0 (0.7)	2.1 (0.8)	2.2 (0.8)	0.004	0.18	0.001	0.006
HDL, mean (SD), mmol/L	1.2 (0.3)	1.3 (0.3)	1.2 (0.3)	1.1 (0.2)	<0.001	<0.001	<0.001	<0.001
TC, mean (SD), mmol/L	5.0 (0.9)	4.9 (0.9)	5.0 (0.9)	5.2 (1.0)	0.01	0.08	0.005	0.048
TG, mean (SD), mmol/L	1.7 (1.2)	1.4 (0.9)	1.8 (1.3)	2.3 (1.6)	<0.001	<0.001	<0.001	<0.001
FBG, mean (SD), mmol/L	5.5 (1.4)	5.3 (1.0)	5.6 (1.5)	6.0 (0.02)	<0.001	<0.001	<0.001	<0.001
SBP, mean (SD), mmHg	123.3 (16.7)	118.9 (15.7)	125.1 (16.2)	133.1 (19.1)	<0.001	<0.001	<0.001	<0.001
DBP, mean (SD), mmHg	78.4 (12.6)	74.1 (11.0)	80.3 (12.6)	86.8 (12.7)	<0.001	<0.001	<0.001	<0.001
Creatinine, mean (SD), mmol/L	66.8 (14.4)	61.4 (12.6)	69.2 (14.5)	77.1 (12.4)	<0.001	<0.001	<0.001	<0.001
SE, mean (SD), diopter	−1.4 (2.1)	−1.6 (2.1)	−1.4 (2.2)	−1.0 (1.8)	0.001	0.01	0.001	0.04
Axial length, mean (SD), mm	23.9 (1.1)	23.9 (1.1)	23.9 (1.1)	23.9 (1.0)	0.46	0.21	0.79	0.71
RBC, mean (SD), 10^12/L	4.7 (0.5)	4.5 (0.4)	4.8 (0.5)	5.0 (0.4)	<0.001	<0.001	<0.001	<0.001
Hemoglobin, mean (SD), g/L	145.2 (17.1)	138.1 (15.1)	148.4 (17)	158.2 (12.2)	<0.001	<0.001	<0.001	<0.001

SD, standard deviation; BMI, body mass index; LDL, low-density lipoprotein cholesterol; HDL, high-density lipoprotein cholesterol; TC, total cholesterol; TG, triglyceride; FBG, fasting blood glucose; SBP, systolic blood pressure; DBP, diastolic blood pressure; SE, spherical equivalence; RBC, red blood cell.

a Never snore vs. occasionally snore.

b Never snore vs. frequently or more severe snore.

c Occasionally snore vs. frequently or more severe snore.

### Total retinal thickness and retinal capillary plexus vessel densities among different snoring groups

Table 2 shows the ocular characteristics in different snoring groups. The whole total retinal thickness was higher in participants who self-reported “occasionally” and “frequently or more severe” snoring compared with those in “never” snoring group (*p* < 0.001 and *p* = 0.001, respectively.) In addition, there was no statistical difference between participants in “occasionally” and “frequently or more severe” snoring groups: (*p* = 0.14) In superficial retina, RCP vessel density of participants with “frequently or more severe” snoring was lower than those who “never” and “occasionally” snore (*p* < 0.001 and *p* < 0.001, respectively.) however, we did not observe a significant difference between participants in “never” and “occasionally” snoring groups (*p* = 0.57) In deep retinal, participants with higher frequency snoring had lower RCP vessel density (all *p* < 0.001) and foveal avascular zone (FAZ) area (all *p* < 0.05). The retinal characteristics of other quadrants were listed in Table 2.

**TABLE 2:** Ocular characteristics of participants in different frequency of self-reported snoring.

Characteristic	Self-reported snoring			*p* value	*P* value^a^	*P* value^b^	*P* value^c^
	never (*n* = 982)	occasionally (*n* = 1,470)	Frequently or more severe (*n* = 170)				
Total retinal thickness, μm							
Whole	319.3 (13.3)	321.5 (14.5)	323.2 (13.9)	<0.001	<0.001	0.001	0.14
Fovea	250.7 (17.2)	254.6 (18.8)	257.3 (17.0)	<0.001	<0.001	<0.001	0.06
ParaFovea	328.0 (13.7)	330.1 (14.9)	331.6 (14.3)	<0.001	<0.001	0.003	0.20
Temporal	318.9 (13.3)	321.4 (14.6)	323.3 (14.0)	<0.001	<0.001	<0.001	0.09
Superior	332.6 (14.4)	334.3 (15.2)	335.3 (15.3)	0.008	0.006	0.03	0.39
Nasal	332.2 (14.6)	334.3 (15.7)	335.8 (14.8)	0.001	0.001	0.006	0.24
Inferior	328.0 (14.2)	330.1 (15.4)	331.8 (14.6)	<0.001	0.001	0.002	0.15
Superficial RCP vessel density, %							
Whole	47.4 (2.7)	47.3 (2.7)	46.6 (2.9)	0.001	0.57	<0.001	<0.001
Fovea	15.3 (5.6)	16.4 (5.8)	16.3 (5.4)	<0.001	<0.001	0.04	0.95
ParaFovea	50.4 (2.9)	50.2 (2.9)	49.4 (3.0)	<0.001	0.27	<0.001	<0.001
Temporal	48.5 (3.7)	48.4 (3.3)	47.6 (3.1)	0.007	0.32	0.002	0.007
Superior	52.2 (3.1)	52.0 (3.5)	51.1 (3.5)	<0.001	0.09	<0.001	0.001
Nasal	49.4 (3.1)	49.4 (3.2)	48.6 (3.1)	0.005	0.71	0.001	0.002
Inferior	51.2 (3.8)	51.1 (3.6)	50.3 (3.6)	0.02	0.70	0.006	0.008
Deep RCP vessel density, %							
Whole	51.6 (3.1)	50.8 (3.1)	49.8 (3.1)	<0.001	<0.001	<0.001	<0.001
Fovea	28.8 (7.0)	29.8 (7.1)	30.4 (6.2)	<0.001	<0.001	0.008	0.35
ParaFovea	54.1 (3.1)	53.1 (3.2)	51.9 (3.1)	<0.001	<0.001	<0.001	<0.001
Temporal	54.0 (3.9)	53.0 (3.4)	51.9 (3.0)	<0.001	<0.001	<0.001	<0.001
Superior	53.9 (3.4)	52.8 (3.9)	51.8 (3.7)	<0.001	<0.001	<0.001	<0.001
Nasal	54.5 (3.0)	53.4 (3.2)	52.4 (3.2)	<0.001	<0.001	<0.001	<0.001
Inferior	53.7 (4.0)	52.8 (3.9)	51.4 (3.5)	<0.001	<0.001	<0.001	<0.001
FAZ area, mm^2^	0.35 (0.1)	0.33 (0.1)	0.31 (0.1)	<0.001	<0.001	<0.001	0.03

RCP, retinal capillary plexus; FAZ, foveal avascular zone.

a Never snore vs occasionally snore.

b Never snore vs frequently or more severe snore.

c Occasionally snore vs frequently or more severe snore.

### Association of snore with retinal thickness

We show the relationships between retinal thickness and snore in Table 3. We added age, sex, BMI, education level, history of diabetes, hypertension, dyslipidemia, smoking and drinking, creatinine level, red blood cell count, and hemoglobin level to the GLM. Compared with the “never” snoring group, the “frequently or more severe” snoring group was associated with thinner total retinal thickness in the whole [*β* = −2.79 (95% CI: −5.27, −0.30)], parafovea [*β* = −2.94 (95% CI: −5.49, −0.38)] and in the four quadrants (Table 3). However, no significant association of retinal thickness was found in the “occasionally” snoring group (Table 3).

**TABLE 3:** Association of self-reported snoring with OCT and OCTA parameters according to frequency.

Characteristic	Self-reported snoring				
	Never adjusted *β* (95%CI)	Occasionally adjusted *β* (95% CI)	*p*-value	Frequently or more severe adjusted *β* (95% CI)	*p*-value
Total retinal thickness					
Whole	0 (Reference)	−1.01 (−2.24, 0.23)	0.11	−2.79 (−5.27, −0.30)	0.03
Fovea	0 (Reference)	−0.62 (−2.20, 0.97)	0.45	−2.22 (−5.41, 0.96)	0.17
ParaFovea	0 (Reference)	−1.11 (−2.39, 0.17)	0.09	−2.94 (−5.49, −0.38)	0.02
Temporal	0 (Reference)	−0.97 (−2.22, 0.27)	0.12	−2.66 (−5.15, −0.16)	0.04
Superior	0 (Reference)	−1.05 (−2.37, 0.28)	0.12	−3.11 (−5.75, −0.46)	0.02
Nasal	0 (Reference)	−1.06 (−2.41, 0.29)	0.12	−2.94 (−5.65, −0.23)	0.03
Inferior	0 (Reference)	−1.12 (−2.44, 0.20)	0.10	−2.73 (−5.37, −0.08)	0.04
Superficial RCP vessel density					
Whole	0 (Reference)	0.10 (−0.14, 0.34)	0.40	−0.71 (−1.19, −0.23)	0.004
Fovea	0 (Reference)	0.10 (−0.41, 0.61)	0.70	−0.88 (−1.90, 0.13)	0.09
ParaFovea	0 (Reference)	0.10 (−0.16, 0.36)	0.44	−0.71 (−1.23, −0.19)	0.007
Temporal	0 (Reference)	0.07 (−0.24, 0.38)	0.67	−0.72 (−1.35, −0.10)	0.02
Superior	0 (Reference)	0.12 (−0.15, 0.40)	0.38	−0.76 (−1.32, −0.20)	0.008
Nasal	0 (Reference)	0.08 (−0.20, 0.37)	0.57	−0.73 (−1.30, −0.16)	0.01
Inferior	0 (Reference)	0.23 (−0.10, 0.56)	0.18	−0.46 (−1.13, 0.20)	0.17
Deep RCP vessel density					
Whole	0 (Reference)	0.17 (−0.09, 0.42)	0.21	0.01 (−0.51, 0.53)	0.97
Fovea	0 (Reference)	0.11 (−0.51, 0.73)	0.73	−0.23 (−1.48, 1.02)	0.71
ParaFovea	0 (Reference)	0.13 (−0.12, 0.38)	0.30	0.03 (−0.47, 0.54)	0.89
Temporal	0 (Reference)	0.17 (−0.13, 0.46)	0.28	0.13 (−0.47, 0.72)	0.68
Superior	0 (Reference)	0.12 (−0.17, 0.42)	0.41	0.04 (−0.55, 0.64)	0.89
Nasal	0 (Reference)	0.07 (−0.18, 0.32)	0.60	0.06 (−0.44, 0.56)	0.81
Inferior	0 (Reference)	0.30 (−0.03, 0.63)	0.07	0.09 (−0.57, 0.75)	0.79

CI, confidence interval; RCP, retinal capillary plexus; FAZ, foveal avascular zone. Adjusted for age, sex, body mass index, education level, diabetes, hypertension, dyslipidemia, smoke, drink, creatinine level, red blood cell count and hemoglobin. Self-reported “never” snore was set as reference.

### Association of snore with retinal capillary plexus characteristics

The relationship of superficial and deep RCP vessel density with snoring groups in different retinal regions were represented in Table 3. After adjusting for the confounders, we found that in superficial RCP, compared with the “never” snoring group, the “frequently or more severe” snoring group was associated with lower vessel density in the whole [*β* = −0.71 (95% CI: −1.19, −0.23)] and parafovea [*β* = −0.71 (95% CI: −1.23, −0.19)] and in other quadrants (Table 3). There was no statistical significance in the “occasionally” snoring group. In the deep RCP vessel density and FAZ area, compared with the “never” snoring group, all snore groups showed insignificant associations (Table 3).

### Subgroup analysis of self-reported snoring according to hypertension

Table 4 showed the associations of self-reported snoring with retinal RCP vessel density stratified by hypertension. After adjusting for the confounders, we did not observe interactions of self-reported with hypertension for both superficial and deep RCP vessel density (all interaction-p > 0.05).

**TABLE 4:** Associations of self-reported snoring with retinal capillary plexus stratified by hypertension.

Retinal capillary plexus	Never	Occasionally		Frequently or more severe		*p* value interaction
		Hypertension (*n* = 416)	Non-hypertension (*n* = 1,054)	Hypertension (*n* = 85)	Non-hypertension (*n* = 85)	
		Adjusted *β* (95% CI)	Adjusted *β* (95% CI)	Adjusted *β* (95% CI)	Adjusted *β* (95% CI)	
Superficial RCP vessel density						
Whole	0 (reference)	−0.06 (−0.60, 0.48)	0.14 (−0.13, 0.40)	−0.82 (−1.61, −0.03)	−0.67 (−1.31, −0.04)	0.79
Fovea	0 (reference)	0.75 (−0.35, 1.84)	−0.07 (−0.64, 0.51)	−0.72 (−2.32, 0.88)	−0.62 (−2.00, 0.75)	0.39
ParaFovea	0 (reference)	−0.22 (−0.81, 0.37)	0.17 (−0.11, 0.46)	−0.86 (−1.73, −0.003)	−0.74 (−1.43, −0.05)	0.46
Temporal	0 (reference)	−0.38 (−0.99, 0.24)	0.17 (−0.19, 0.54)	−1.05 (−1.95, −0.15)	−0.69 (−1.56, 0.18)	0.46
Superior	0 (reference)	−0.01 (−0.64, 0.62)	0.14 (−0.17, 0.45)	−1.00 (−1.93, −0.08)	−0.63 (−1.37, 0.11)	0.85
Nasal	0 (reference)	−0.36 (−1.01, 0.29)	0.19 (−0.12, 0.50)	−0.74 (−1.69, 0.21)	−0.90 (−1.65, -0.15)	0.12
Inferior	0 (reference)	−0.13 (−0.82, 0.56)	0.30 (−0.08, 0.68)	−0.66 (−1.67, 0.34)	−0.49 (−1.40, 0.41)	0.69
Deep RCP vessel density						
Whole	0 (reference)	0.36 (−0.23, 0.96)	0.10 (−0.19, 0.38)	0.47 (−0.40, 1.34)	−0.28 (−0.97, 0.40)	0.53
Fovea	0 (reference)	1.03 (−0.25, 2.31)	−0.14 (−0.86, 0.57)	0.25 (−1.62, 2.13)	−0.10 (−1.82, 1.61)	0.38
ParaFovea	0 (reference)	0.26 (−0.34, 0.85)	0.09 (−0.18, 0.36)	0.51 (−0.36, 1.37)	−0.32 (−0.97, 0.33)	0.34
Temporal	0 (reference)	0.28 (−0.29, 0.85)	0.12 (−0.23, 0.47)	0.42 (−0.40, 1.25)	−0.13 (−0.97, 0.71)	0.66
Superior	0 (reference)	0.34 (−0.36, 1.04)	0.05 (−0.27, 0.37)	0.64 (−0.39, 1.66)	−0.35 (−1.12, 0.42)	0.54
Nasal	0 (reference)	0.06 (−0.53, 0.65)	0.05 (−0.22, 0.33)	0.39 (−0.48, 1.26)	−0.20 (−0.85, 0.45)	0.43
Inferior	0 (reference)	0.35 (−0.36, 1.06)	0.27 (−0.10, 0.65)	0.58 (−0.45, 1.61)	−0.33 (−1.22, 0.56)	0.33

CI, confidence interval; RCP, retinal capillary plexus. Adjusted for age, sex, body mass index, education level, diabetes, dyslipidemia, smoke, drink, creatinine level, red blood cell count and hemoglobin. Self-reported “never” snore was set as reference.

## Discussion

To our knowledge, this is the first study assessing the association of self-reported snoring with retinal thickness and RCP vessel density in a large community-based population using OCT and OCTA. We found that snoring was associated with thin retinal thickness and low superficial RCP vessel density. Unfortunately, data on clinical diagnosis of OSA were not available in present study. Given snoring is an early symptom of OSA, effect of OSA-related pathology on retinal health could not be rule out from the present study.

We found compared with non-snorers, subjects who frequently or more severe snored had thinner total retinal thickness. Snoring is considered a common symptom and early marker of OSA, and previous studies have reported that OSA is associated with neuro-ophthalmologic diseases (Wong and Fraser, 2019). Ching et al. (Lin et al., 2013) found that the hazard ratio of open-angle glaucoma in subjects with OSA was 1.67 higher than those of the comparison group in the 5-years follow-up period. Fan et al. (2019) also reported that severe OSA is significantly associated with the progressive decrease in RNFL thickness in patients with glaucoma. Furthermore, several studies had found that retinal thickness is decreased in patients with different severities of OSA (Casas et al., 2018; Kısabay Ak et al., 2020; Naranjo-Bonilla et al., 2021). However, obtaining an accurate diagnosis of OSA is sometimes difficult. Polysomnography is considered the gold-standard in clinical practice and requires patients to complete an overnight examination. Therefore, snoring assessment is a more intuitive and convenient method with greater economic benefits for large-scale clinical surveys. In this study, we also observed that people with snoring had significant decreased total retinal thickness, which may share the same pathogenic mechanism with OSA. Hypoxia caused by intermittent upper airway obstruction during sleep results in an increase in PaCO_2_ and a decrease in PaO_2_ (Prabhakar et al.,2020). This paroxysmal vascular insufficiency may impair optic nerve perfusion and oxygenation, and result in further optic neuropathy (Lin et al., 2013).

We found that compared with people who do not snore, subjects who self-reported that they “frequently or more severe” snored had lower superficial RCP vessel density. Some studies have reported that people who snore have a higher risk of cardiovascular diseases and adverse events than those in comparison. (Nagayoshi et al., 2012; Li et al., 2020). Muskaan et al. (Behl et al., 2014) observed that snorers had lower flow-mediated dilation of side branch vessels on ultrasound. Arrigo et al. (Cicero et al., 2016) found the arterial stiffness markers (pulse wave velocity and augmentation index) were significantly higher in those who snore than in controls. Li et al. (2012) investigated the carotid artery using ultrasonography in people who are self-reported snorers and imaging showed that the maximum intima-media thickness increased not only in common but also in bifurcation carotid arteries in those who snore. Furthermore, the increased thickness odds ratios of those who habitually snore were 1.71 and 3.63, respectively. In addition, current studies mostly focus on the cardiovascular field, while microvascular changes have not yet been reported in snorers.

Several OCTA studies have reported decreasing retinal vessel density in OSA patients (Ucak and Unver, 2020; Ye et al., 2021; Çolak et al., 2021; Ava et al., 2022). Our research supported these findings as we also observed lower superficial RCP vessel density in those who snore according to self-reported information. In previous studies, snoring was only considered a marker of OSA (Rosen et al., 2019; Li et al., 2020). Thus, our findings expanded the relationship between snoring and vascular impairment in the community-based population. Potentially, the reduced RCP vessel density in subjects who snore may be explained by sympathetic activation, oxidative stress and intimal injury caused by hypoxia (Behl et al., 2014; Prabhakar et al.,2020). However, further research is required to explore the internal mechanism.

In addition, we did not find any association between snoring and deep RCP vessel density. Amatoury et al. (2006) and Howitt et al. (2007) found that in rabbits, the carotid artery wall and the artery lumen could be affected by the vibration of snoring. The mechanical vibration may not propagate to the capillaries. This hypothesis may explain the association only presented in the superficial RCP, rather than in deep RCP. Compared with the deep retina, the superficial retina had branch blood vessels with larger diameters and was more vulnerable to the effect of snoring.

The associations of the both superficial and deep RCP vessel density with self-reported snoring were consistent across the subgroup based on hypertension. Although previous study had found the effect of hypertension to OCTA metrics (Peng et al., 2020), this interactions seem to be absent in our study. This might attribute to regularly antihypertensive drugs taking. Participants in our study had five types of antihypertensive agents: Betablockers, Ca antagonists, diuretic, angiotensin converting enzyme inhibitor, and angiotensin receptor blockers, and all subjects reported a once-daily intake.

The major strength of our study was the simultaneous assessment of retinal structure and microcirculation based on a self-reported snoring assessment. However, some limitations also apply. First, this is a single cross-sectional study, the internal causal relationships would be indistinguishable and further multicenter study is needed to expand the results to other population. Second, self-reported snoring information was collected from the questionnaire, so there may be bias and misclassification between actual and self-reported snoring. Third, we could not distinguish those with OSA from those who snore, which requires complex and time-consuming examination. Thus, the possibility would not be excluded that OSA, as a confounder, might have affected the association. However, the major purpose of this study was to analyze the association of retinal parameters with easily accessible self-reported snoring indicator, and its benefit ratio and enforceability could be expected.

## Conclusion

In conclusion, we found that snoring was associated with decreased retinal thickness and lower RCP vessel density. Our study suggests that, despite the insufficiency of clinical diagnosis of OSA, self-reported snoring information was also a marker for retinal structure and microcirculation impairment. This research also supported the use of OCT and OCTA to help evaluate the severity of peripheral nerve and systemic microvascular injury in people who snore.

## Data Availability

The raw data supporting the conclusion of this article will be made available by the authors, without undue reservation.
